# The association of endothelial cell signaling, severity of illness, and organ dysfunction in sepsis

**DOI:** 10.1186/cc9290

**Published:** 2010-10-13

**Authors:** Nathan I Shapiro, Philipp Schuetz, Kiichiro Yano, Midori Sorasaki, Samir M Parikh, Alan E Jones, Stephen Trzeciak, Long Ngo, William C Aird

**Affiliations:** 1 Department of Emergency Medicine, Beth Israel Deaconess Medical Center,1 Deaconess Road CC2-W, Boston, MA 02215, USA; 2 Center for Vascular Biology Research, Beth Israel Deaconess Medical Center, 99 Brookline Avenue, Boston, MA 02215, USA; 3 Division of Nephrology, Beth Israel Deaconess Medical Center, 99 Brookline Street, Boston, MA 02215, USA; 4 Division of General Medicine, Department of Medicine, Beth Israel Deaconess Medical Center, 1309 Beacon Street, Office 203, Brookline, MA 02446, USA; 5 Department of Emergency Medicine Carolinas Medical Center, 1000 Blythe Blvd, Charlotte, NC 28203, USA; 6 Cooper University Hospital, One Cooper Plaza, Camden, NJ 08103, USA

## Abstract

**Introduction:**

Previous reports suggest that endothelial activation is an important process in sepsis pathogenesis. We investigated the association between biomarkers of endothelial cell activation and sepsis severity, organ dysfunction sequential organ failure assessment (SOFA) score, and death.

**Methods:**

This is a prospective, observational study including adult patients (age 18 years or older) presenting with clinical suspicion of infection to the emergency department (ED) of an urban, academic medical center between February 2005 and November 2008. Blood was sampled during the ED visit and biomarkers of endothelial cell activation, namely soluble fms-like tyrosine kinase-1 (sFlt-1), plasminogen activator inhibitors -1 (PAI-1), sE-selectin, soluble intercellular adhesion molecule (sICAM-1), and soluble vascular cell adhesion molecule (sVCAM-1), were assayed. The association between biomarkers and the outcomes of sepsis severity, organ dysfunction, and in-hospital mortality were analyzed.

**Results:**

A total of 221 patients were included: sepsis without organ dysfunction was present in 32%, severe sepsis without shock in 30%, septic shock in 32%, and 6% were non-infected control ED patients. There was a relationship between all target biomarkers (sFlt-1, PAI-1, sE-selectin, sICAM-1, and sVCAM-1) and sepsis severity, *P *< 0.05. We found a significant inter-correlation between all biomarkers, including the strongest correlations between sFlt-1 and sE-selectin (r = 0.55, *P *< 0.001), and between sFlt-1 and PAI-1 (0.56, *P *< 0.001). Among the endothelial cell activation biomarkers, sFlt-1 had the strongest association with SOFA score (r = 0.66, *P *< 0.001), the highest area under the receiver operator characteristic curve for severe sepsis of 0.82, and for mortality of 0.91.

**Conclusions:**

Markers of endothelial cell activation are associated with sepsis severity, organ dysfunction and mortality. An improved understanding of endothelial response and associated biomarkers may lead to strategies to more accurately predict outcome and develop novel endothelium-directed therapies in sepsis.

## Introduction

Despite recent advances in biomedical research, sepsis remains an important medical challenge. An estimated 750,000 cases of severe sepsis are diagnosed each year in the United States alone [1], incurring health care costs of $16.7 billion annually [2]. One major potential shortcoming of prior therapeutic approaches in sepsis is the attempt to target one specific pathway, component, or cytokine involved in the host response; however, the host response in sepsis is coordinated across multiple pathways including inflammation, coagulation, metabolism and tissue hypoxia. An important goal in sepsis research is to develop a more detailed understanding of the mechanisms underlying the host response to infection, with the expectation that such studies will yield novel insights into potential diagnostic and therapeutic targets.

There is increasing evidence that the endothelium plays a central and pathogenic role in sepsis. Endothelial cells are diverse in function and highly responsive to their extracellular environment (reviewed in [3]). When exposed to certain agonists, such as lipopolysaccharide, cytokines, chemokines or growth factors, endothelial cells become activated. The activation state is manifested by enhanced permeability, increased leukocyte adhesion, a shift in the hemostatic balance towards a procoagulant phenotype, and altered regulation of vasomotor tone. Collectively, these changes likely evolved as an adaptive host response to extravascular pathogens, allowing for increased blood flow to the area of insult, local efflux of plasma proteins and leukocytes, and sequestering of the infection. This activated state may be considered dysfunctional when an overactive endothelium disturbs the homeostatic state instead of restoring it, representing a net liability to the host. In this context, endothelial dysfunction typically involves some combination of increased leukocyte adhesion and transmigration, increased permeability, a shift in the hemostatic balance towards the procoagulant side and an alteration in vasomotor tone. In sepsis, endothelial activation and dysfunction are critical determinants of the host response and, thus, represent a unifying explanation for the complex sepsis pathophysiology, as well as an attractive target for systemic therapy.

**Figure 1:**
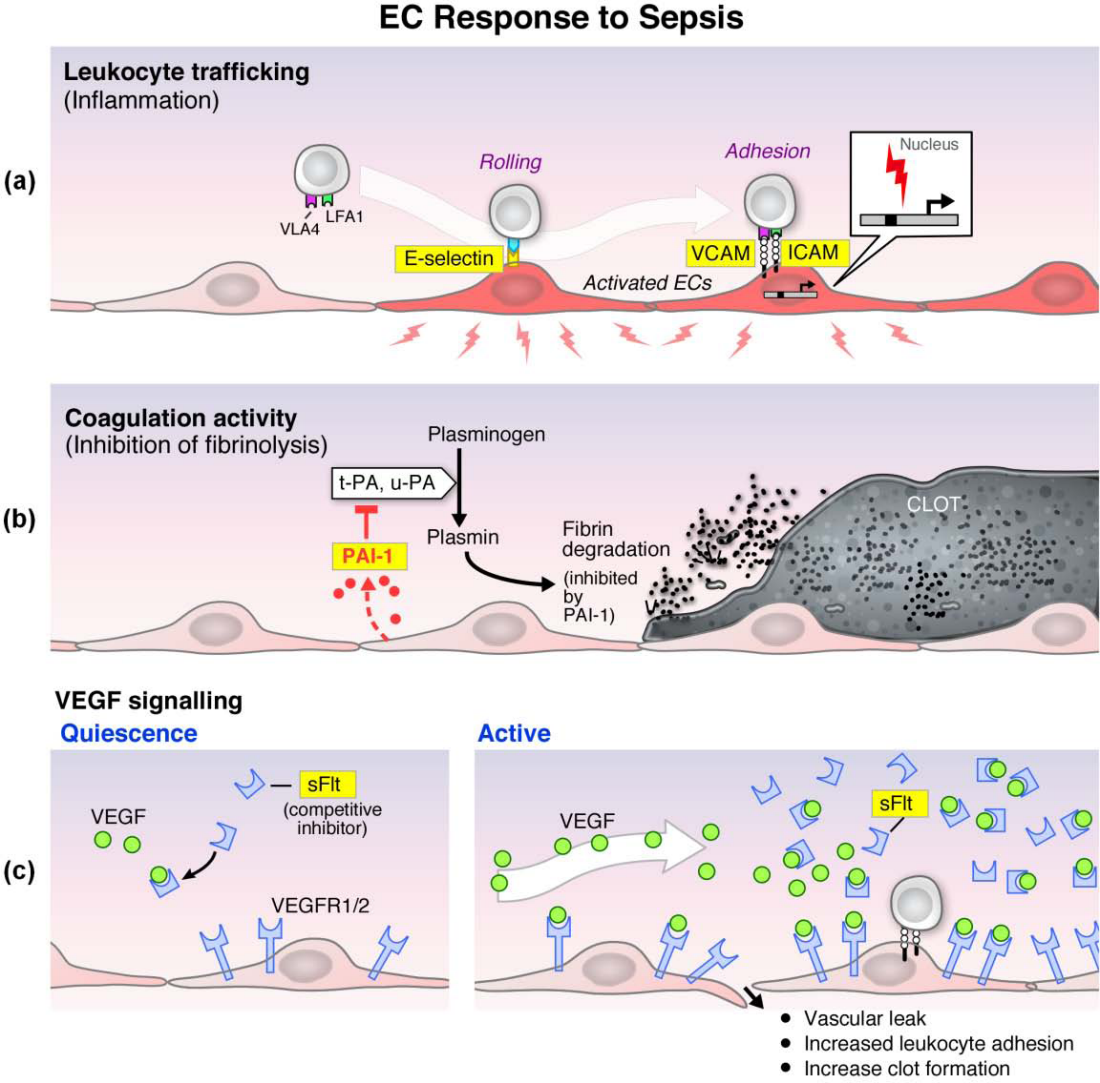
**Endothelial cell response in sepsis**. **(a) **Leukocyte trafficking. Activated endothelial cells (red-colored cells) express increased levels of E-selectin, P-selectin, intercellular adhesion molecule (ICAM)-1 and vascular cell adhesion molecule (VCAM)-1 (all but P-selectin are shown). Upregulation of E-selectin, ICAM-1 and VCAM-1 are mediated at a transcriptional level (activation signal and promoter with transcriptional start site are shown in the inset). E selectin induces rolling of circulating leukocytes. VCAM-1 and ICAM-1 induce firm adhesion of leukocytes by binding to very late antigen 4 (VLA4) and leukocyte function antigen LFA1, respectively. Following firm adhesion, leukocytes transmigrate through and/or between endothelial cells into the underlying tissue (not shown). In sepsis, E-selectin, ICAM-1, and VCAM-1 are cleaved from the cell surface and circulate as a soluble form of the receptor. Circulating levels are indirect measures of the degree of endothelial activation. **(b) **Hemostasis. Activated endothelial cells undergo a net shift in hemostatic balance towards the procoagulant side, leading to local clot formation. During fibrinolysis tissue-type plasminogen activator (t-PA) and urokinase-type plasminogen activator (u-PA) mediate the conversion of plasminogen to plasmin. Plasmin, in turn, proteolytically degrades fibrin. Activated endothelial cells express increased levels of plasminogen activator inhibitor (PAI-1), which inhibit the activity of t-PA and u-PA, thus accentuating the procoagulant state. **(c) **Vascular endothelial growth factor (VEGF) signaling. Under normal conditions (quiescence), VEGF signaling plays a critical role in homeostasis. VEGF binds to two receptors on endothelial cells, VEGF receptor (VEGFR) 1 and 2. VEGFR1 is also known as Flt-1. In sepsis (*activated *state), circulating levels of VEGF are increased. Elevated VEGF signaling, in turn, leads to increased vascular leak, leukocyte adhesion/trafficking, and clot formation. Sepsis is also associated with increased circulating levels of a soluble form of VEGFR1 (sFlt-1). sFlt-1 binds VEGF in the blood, thus acting as a competitive inhibitor of VEGF signaling in endothelial cells. Sepsis-mediated induction of sFlt-1 may represent a critical component of the host anti-inflammatory response.

The aim of the present study was to assay a broad range of endothelial markers in a large sample of human patients at the time of emergency department (ED) presentation with the goal of gaining further insights into the activation state of the endothelium in different stages of sepsis. To that end, we have measured circulating levels of soluble leukocyte adhesion molecules (soluble vascular cell adhesion molecule (VCAM)-1, soluble intercellular adhesion molecule (ICAM-1) and sE-selectin; procoagulant/antifibrinolytic mediators (plasminogen activator inhibitors (PAI)-1); and a marker of vascular endothelial growth factor (VEGF) signaling (sFlt-1) (reviewed in Figure 1) in 221 septic patients with varying degrees of severity. We analyzed the relationships between the biomarkers of endothelial cell activation and sepsis severity, inflammatory response, organ dysfunction, and mortality. An improved understanding of the endothelial cell response in sepsis may suggest avenues for diagnostic platforms, and could also delineate new strategies for identifying patients with endothelial cell dysfunction that may be particularly responsive to therapies targeted to restore endothelial health.

## Materials and methods

### Design and population

This was a prospective, cohort study of a convenience sample of adult patients (age 18 years or older) presenting to the ED with suspected infection. Suspected infection was defined as a clinical suspicion of an infectious etiology as assessed by the treating clinician, and determined by interviewing the treating physician to determine if infection was suspected based on the ED work-up including the results from history, physical exam, laboratory and diagnostic testing. The population was selectively enrolled to achieve a relatively even distribution of different sepsis severities. A sample of non-infected ED *control *patients was also assembled by identifying adult ED patients without evidence of infection during presentation. The study period was between February 2005 and November 2008. There were 221 patients enrolled in the study with 189 patients enrolled *de novo*, and 32 patients co-enrolled with another protocol [4]. The setting was Beth Israel Deaconess Medical Center (BIDMC), Boston, an urban teaching hospital. The study was approved by the hospital ethics board, and written informed consent was obtained.

### Collection of clinical covariates

In order to characterize the population, relevant components of demographics, history, co-morbid diseases, suspected source of infection, vital sign information, physical exam findings, and the results of laboratory and radiologic testing were collected. The Charlson comorbidity index, a well established methodology to quantify co-morbid disease burden, was calculated for each patient [5].

### Biomarker analysis

All subjects received a blood draw while in the emergency department. Samples were drawn in EDTA tubes, centrifuged at 2,500 × g at 4°C, and frozen at -80°C within one hour of collection. Plasma was assayed for sE-selectin, sICAM-1, sVCAM-1, and PAI-1 as a multiplex panel using the human cardiovascular-1 panel (Millipore, Billerica, MA, USA) and Interleukin-6 (IL-6) using the human cardiovascular-3 panel (Millipore) on the Luminex 200 instrument (Millipore). The sFlt-1 assays were performed using Quantikine ELISA kits (R&D systems, Minneapolis, MN, USA). All assays were performed in duplicate and the average levels were used for analysis.

### Septic shock subset with daily blood draws

Between January 2007 and January 2009, patients in our study with septic shock received additional blood draws every 24 hours for the first three days - a total of 52 patients were enrolled in this subset. This sub-study was performed to assess the changes in the circulating biomarkers of endothelial cell activation over time.

### Outcomes assessment

#### Sepsis severity classification

Sepsis severity was characterized according to a modified version of the ACCP/SCCM sepsis syndromes [6]. We have previously published on the details and validity of these modified definitions [7]. Patients were characterized into one of the following groups: non-infected ED patients, sepsis, severe sepsis, or septic shock. For assessment of organ dysfunction, we used the SOFA score, and for additional severity of illness assessment [8], we used the Acute Physiologic And Chronic Health Evaluation (APACHE)-II score [9] based on the worst values over the first 24 hours, as originally described. Serum lactate levels were used as another severity of illness marker [10] and were either obtained as part of routine clinical care, or assayed using a point-of-care i-stat device (Abbott Point-of-Care, Princeton, NJ, USA). We have previously affirmed the concordance of these two methods [11].

#### Sepsis severity classification

Non-infected ED patients were defined as patients presenting to the ED without a clinical suspicion of infection. Sepsis was comprised ED patients with suspected infection with or without systemic inflammatory response syndrome (SIRS). The decision to combine these groups (with and without SIRS) was based on our previous publication demonstrating no mortality difference based on SIRS criteria alone so that *severity *is equivalent [7,12]. Severe Sepsis was defined as sepsis with concomitant organ dysfunction defined by meeting one or more of the following organ dysfunction definitions; central nervous system: new altered mental state and/or new onset of GCS < 15; respiratory: any mechanical ventilation, supplemental oxygen required to maintain oxygen saturation > 95%, and/or respiratory rate > 24 beats per minute; cardiovascular: any vasopressor use, SBP < 90 mmHg after 20 mL/kg bolus; renal: urine output < 0.5 mL/kg/hr, or creatinine > 50% of baseline or > 2. mg/dl if baseline is unknown; hepatic: AST/ALT > 80 (new); hematopoietic: platelet count < 100,000 and/or PT/PTT > 50% of normal; or metabolic: lactate > 2.5 mmol/l. Septic shock was defined as sepsis plus hypotension (SBP < 90 mmHg after 20 to 30 cc/kg fluid challenge). The sepsis severity was assessed on presentation and daily for the first 72 hours or until hospital discharge, assigning a patient to the worst syndrome achieved on a daily basis.

#### Organ dysfunction

The sequential organ failure assessment (SOFA) score was used to assess organ failures [8]. The SOFA score is designed to identify morbidity and individualizes the dysfunction or failure of each organ system. It has been established as a valid predictor for both initial and serial assessments [13-15]. The SOFA score was assessed on presentation and then daily for the first 72 hours or until hospital discharge.

#### Other Inflammatory response and Illness severity markers

IL-6 level was used as a prototype marker of inflammatory response. APACHE-II score was used as a secondary assessment of severity based on worst vital signs, as originally described [9]. This score has been validated as an assessment tool for risk-stratification, and was utilized to characterize disease severity. While some of the baseline variables make it a score that is not necessarily responsive to acute disease state, its prognostic ability has been well established. The APACHE-II score was assessed on presentation, and then daily for the first 72 hours or until hospital discharge. Mortality was defined by hospital discharge disposition.

### Statistical analysis

Means with standard deviations, medians with interquartile ranges, and proportions were used for descriptive statistics, as appropriate. To analyze the association between the biomarkers of endothelial cell activation and sepsis severity, we used generalized linear modeling. Next, we calculated Spearman rank correlation coefficients to assess the bivariable association among the biomarkers. We display the graphs with a regression line and reported the calculated Spearman correlation coefficient (r-value) along with the associated *P*-value. We performed a similar analysis between the target biomarkers and organ dysfunction (SOFA score), the inflammatory response marker IL-6, and APACHE-II score. Due to non-normal distribution, SOFA score was log transformed throughout the analysis. As a comparator, we also examined the correlation of IL-6 and serum lactate with SOFA score. Next, to compare the strength of association between each of the biomarkers and organ dysfunction, we standardized each of the biomarkers values through the following formula: ((biomarker - biomarker mean)/biomarker SD). We then used a linear regression model and adjusted for age, gender, and co-morbid illness burden (Charlson score). We report the beta coefficient with standard error as well as the adjusted r-squared value for each biomarker model. We also tested multi-marker models to determine the value of combinations of biomarkers. To assess the clinical predictive ability of the biomarkers, we calculated the area under the receiver operating characteristic curve (AUC) with 95% confidence interval for each biomarker to predict the outcomes of severe sepsis (including septic shock) within 72 hours and in-hospital mortality. The AUCs were compared nonparametric approach [16].

Finally, for the subset analysis of biomarkers from patients with septic shock collected daily over the first 72 hours of hospitalization, we used a linear mixed effects model to estimate the differences in biomarkers between survivors and non-survivors over time. The linear mixed-effects model took into account the multiple measurements (at 0, 24, 48, 72 hours) of biomarkers and outcomes and used compound symmetry variance-covariance structure to account for the within-subject correlation.

## Results

### Population characteristics

There were a total of 221 patients enrolled with a mean age of 58 (SD +/- 19) years; 52% were male, 76% Caucasian, and there was a high co-morbid burden: diabetes (26%), cancer (20%) and chronic heart failure in 13% (Table 1). On admission, sepsis without organ dysfunction was present in 32%, severe sepsis without shock in 30%, and septic shock in 32%. Six percent were non-infected ED patients who were used as controls. The overall in-hospital mortality in the population was 7.7% (13/221), and 42% (84/221) of patients were admitted to the intensive care unit (ICU).

**Table 1:** Patient characteristics

Parameters	Overall
	*n *= 221
**Demographics**	
Age median, mean (SD)	57, 58 (19)
Race: white n (%)	169 (76%)
african-american	28 (13%)
Other	24 (11%)
Female gender n (%)	115 (52%)
	
**Comorbidities **n (%)	
COPD	16 (7%)
Chronic Heart failure	29 (13%)
Diabetes	63 (28%)
Cancer	45 (20%)
	
**Sepsis Syndrome **n(%)	
Non-infected ED patients	14 (6%)
Sepsis without organ dysfunction	70 (32%)
Severe Sepsis without shock	66 (30%)
Septic Shock	71 (32%)
	
**Severity of Disease**, median, mean (SD)	
SOFA score	2, 3 (4)
APACHE score	11, 12 (8)
Lactate (mg/dL)	1.5, 2.1(1.7)
	
Marker levels on admission* median, mean (SD)	
Eselectin (ng/mL)	49.3, 67.5 (55.4)
VCAM-1 (ng/mL)	1,120, 1,411 (1,316)
ICAM-1 (ng/mL)	176, 224 (151)
PAI-1 (ng/mL)	40.9 64.6 (644)
sFlt-1 (pg/mL)	118, 194 (224)

### Endothelial cell activation and sepsis severity

**Figure 2:**
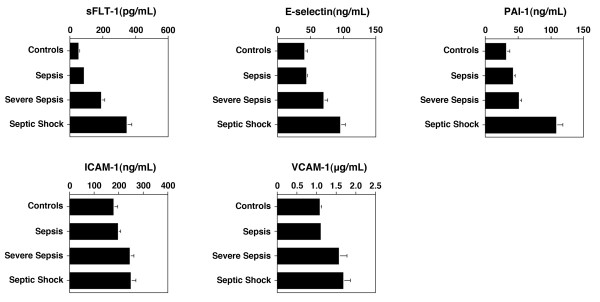
**Median biomarker levels by sepsis syndrome severity**. Median biomarker levels with standard error bars are shown. There was a statistically significant association between biomarker levels and sepsis severity (worst sepsis syndrome within 72 hours) for sFlt-1 (P < 0.001), PAI-1 (*P *< 0.001), sE-selectin (*P *< 0.001), sICAM-1 (*P *< 0.05), and sVCAM-1 (*P *< 0.04).

We found an association between biomarker levels and sepsis severity (worst sepsis syndrome within 72 hours) for sFlt-1 (*P *< 0.001 for trend across groups), PAI-1 (*P *< 0.001), sE-selectin (*P *< 0.001), sICAM-1 (*P *< 0.05), and sVCAM-1 (*P *< 0.04) (Figure 2). The most significant increases were found in median sFlt-1 levels, which ranged from 41 ng/ml (IQR 31 to 51) in non-infected controls to 243 ng/ml (IQR 137 to 449) in septic shock; and, in PAI-1 which ranged from 25.3 ng/ml (IQR 17.6 to 36.8) to 76.7 ng/ml (IQR 49.4 to 136).

### Evidence of endothelial cell activation

**Figure 3:**
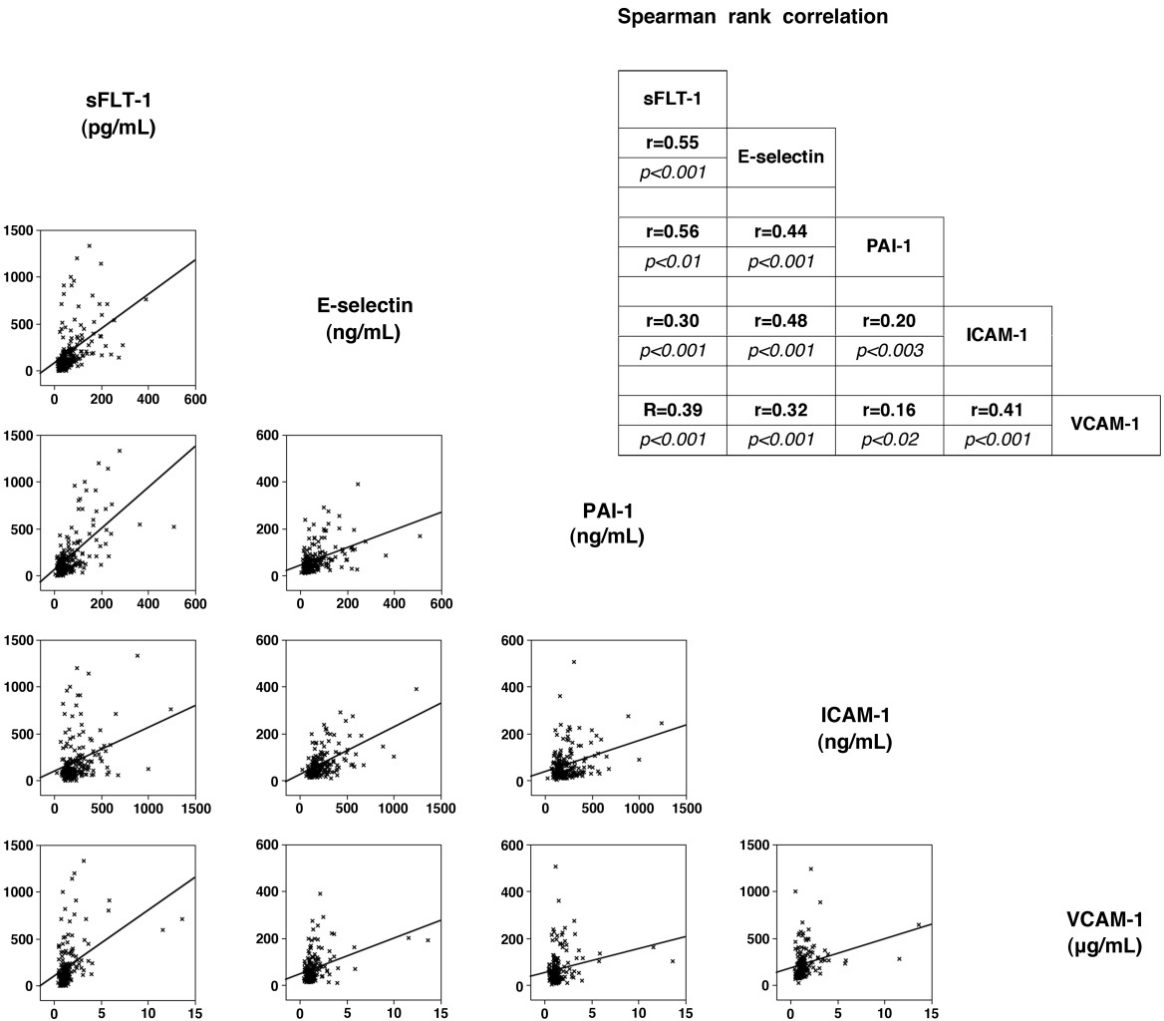
**Correlation of biomarkers of endothelial cell activation with each other**. There correlation graphs and Spearman rank correlation coefficients (r value) are shown along with statistical significance of the correlation.

To assess whether there was evidence of endothelial cell activation in the response to infection, we correlated the selected biomarkers which individually represent various components of the endothelial cell signaling pathway. Using a Spearman rank correlation coefficient, we found a significant correlation between all biomarkers (sFlt-1, PAI-1, sE-selectin, sICAM-1, and sVCAM-1) (Figure 3). The strongest correlations were between sFlt-1 and sE-selectin (r = 0.55, *P *< 0.001) and sFlt-1 and PAI-1 (0.56, *P *< 0.001).

### Endothelial cell activation biomarkers and organ dysfunction

**Figure 4:**
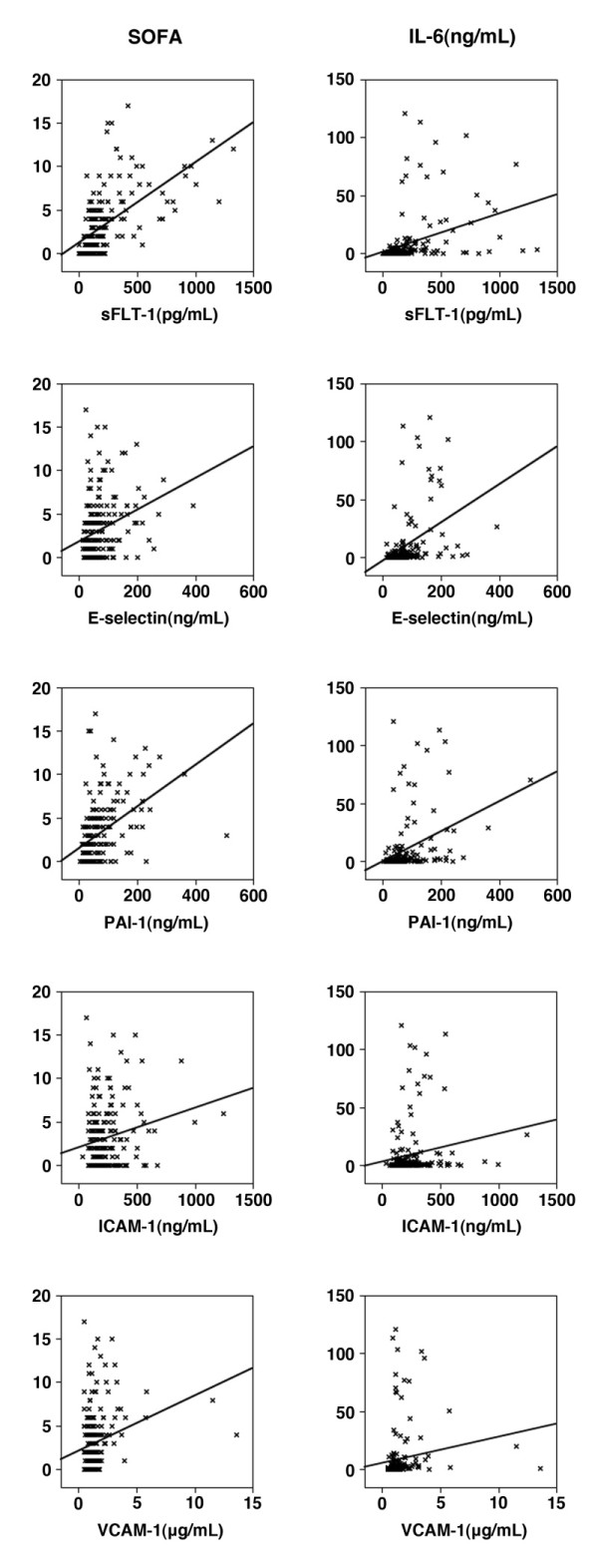
**Correlation of biomarkers of endothelial cell activation with SOFA score and IL-6**. The correlation graphs and Spearman rank correlation coefficients (r value) are shown along with statistical significance of the correlations.

To assess the association of endothelial cell related biomarkers with organ dysfunction, we analyzed the correlation between the endothelial related biomarkers with SOFA score in the ED. All biomarkers were significantly correlated with the concurrent SOFA score (Figure 4). Of note, sFlt-1 was highly correlated (r = 0.66, *P *< 0.001) with SOFA score, and compared favorably in predicting SOFA score to other common biomarkers of inflammation such as IL-6 (r = 0.45) and lactate (r = 0.43). In addition, biomarker levels at the time of presentation correlated with SOFA score at 24 hours: sE-selectin (0.37), sFlt-1 (0.64), sVCAM-1 (0.22), and PAI-1 (0.51), *P *< 0.001 for all comparisons; except sICAM-1 (0.13), *P *= 0.08.

### Endothelial cell activation biomarkers and inflammation

We used circulating IL-6 concentrations as a read-out of the pro-inflammatory response. There was a notable association between the biomarker levels of endothelial activation and IL-6 (Figure 4). Here, sFlt-1 had a particularly strong correlation with IL-6 (r = 0.62, *P *< 0.001).

### Endothelial cell activation biomarkers and other severity of illness markers

Endothelial cell activation markers correlated with two independent markers of disease severity, lactate and APACHE-II scores. There was a significant correlation using Spearman rank between the target biomarkers and APACHE-II score: sFlt-1 (r = 0.58, *P *< 0.01), pai1 (0.46, *P *< 0.01), sE-selectin (0.33, *P *< 0.01), sICAM-1 (0.15, *P *< 0.03), and sVCAM-1 (0.25, *P *< 0.01). These results compare favorably to the r-value for the correlation between classic biomarkers such as lactate with APACHE-II (0.38) and IL-6 with APACHE-II (0.43). There was a significant association between the endothelial related biomarkers and lactate level: sFlt-1 (0.51, *P *< 0.01), PAI-1 (0.40, *P *< 0.01), sE-selectin (0.33, *P *< 0.01), sICAM-1 (0.23, *P *< 0.01), and sVCAM-1 (0.20, *P *< 0.01). As a comparator, IL-6 correlation coefficient with lactate was 0.44.

### Biomarker association with organ dysfunction adjusted for age, gender, and co-morbid illness burden

We analyzed the association of the biomarkers with organ dysfunction (log SOFA score) with linear regression models adjusted for age, gender, and co-morbid illness burden (Table 2) using beta coefficients standardized to a 0 to 100 scale to allow equal comparison. We report the models testing one marker at a time (Table 2). We then checked to see if model fit (measured by adjusted R^2^) would be improved by any combination of multiple markers in the model. Interestingly, once sFlt-1 was included in the models, no additional marker becomes significant if added. The R^2 ^value in the adjusted model for sFlt-1 alone was 0.46, and adding any second marker did not improve the model fit above this level. Additionally, and there was no other combination of two or more markers that exceeds the R^2 ^of the model with sFlt-1 alone, including adding IL-6 and lactate as eligible covariates. Thus, the marker sFlt-1 appears to have the strongest association with organ dysfunction.

**Table 2:** Association of individual biomarkers with organ dysfunction, adjusted for age, gender, and comorbid burden

	*Organ dysfunction * *(log tranformed SOFA score)*			
	
***Biomarker***	***Std. beta***	***SE***	*P*-value	Model adj. r^2^
**sFlt-1**	0.39	0.05	< 0.001	0.46
**PAI-1**	0.29	0.05	< 0.001	0.38
**E-selectin**	0.20	0.05	< 0.001	0.33
**ICAM-1**	0.11	0.05	< 0.04	0.29
**VCAM-1**	0.15	0.05	< 0.003	0.30

Table 2 shows the results from each individual biomarker incorporated into a linear regression model (one marker per model) with outcome SOFA, adjusted for age (years), gender, and co-morbid burden (charlson index). Thus, each line represents its own model: Expected log SOFA = intercept + α (Biomarker) + β(Age) + χ (gender) + δ (Charlson). The biomarkers are standardized [(biomarker - biomarker mean)/SD] so the beta estimates are comparable. Each biomarker showed a statistically significant association with SOFA score. sFlt-1 demonstrates the largest beta estimate which is also supported by an adjusted r-squared in the model of 0.46.

### Biomarkers as predictors of severe sepsis and mortality

To further assess the clinical accuracy of the different markers, we report the area under the receiver operating characteristic curve for the ability of the biomarker drawn on ED presentation to predict two clinical outcomes: 1) severe sepsis (including septic shock as cardiovascular dysfunction) within 72 hours; and, 2) in-hospital mortality (Table 3). Again, sFlt-1 performed with the highest accuracy, and has a higher AUC (0.82; 95% CI 0.76 to 0.88) for severe sepsis when compared to all other endothelial related biomarkers (*P *< 0.05). For the outcome of in-hospital mortality, sFlt-1 had an AUC of 0.91 (0.87 to 0.95), and was also higher (*P *< 0.05) than the AUC for all other markers (Table 3).

**Table 3:** Area under the curve for each biomarker as a predictor of severe sepsis and death

	*Outcome*			
	
	***Severe Sepsis***		***Death***	
		
***Biomarker***	***AUC***	***95% CI***	***AUC***	***95% CI***
**sFlt-1**	0.82*	*0.76 to 0.88*	0.91*	*0.87 to 0.95*
**PAI-1**	*0.69*	*0.62 to 0.76*	*0.74*	*0.60 to 0.88*
**Eselectin**	*0.71*	*0.64 to 0.78*	*0.65*	*0.49 to 0.82*
**Icam**	*0.61*	*0.53 to 0.69*	*0.72*	*0.57 to 0.87*
**Vcam**	*0.60*	*0.52 to 0.69*	*0.57*	*0.35 to 0.79*

*the area under the curve for sFlt-1 in predicting both severe sepsis (includes patients with septic shock) and mortality was significantly greater than all other AUC values, *P *< 0.01.

### Performance of daily markers in septic shock

**Figure 5:**
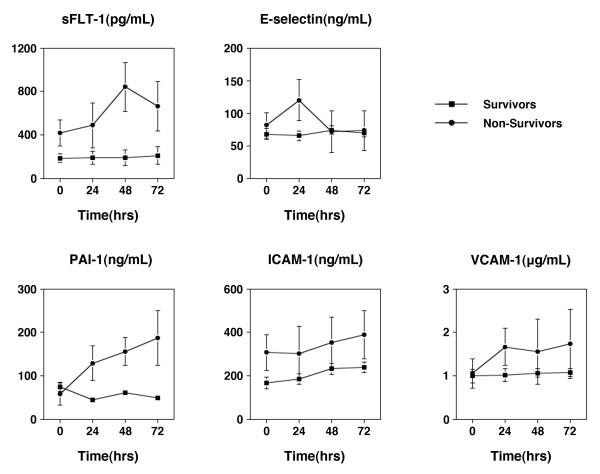
**Comparison of biomarkers levels for survivors and non-survivors in septic shock subset**. Shown here are the biomarker levels for the subset (*n *= 52) of patients with septic shock who had serial blood draws at 0, 24, 48 and 72 hours and were used to compare biomarker levels between survivors (*n *= 43) and non-survivors (*n *= 9). Using a linear mixed effects model, adjusting for age, gender, and co-morbid burden, we found the following estimated mean differences in biomarker levels over time comparing the non-survivors to survivors: sFlt-1 366 pg/mL (95% CI: 218 to 514, *P *< 0.01); PAI-1 63.2 ng/ml (38.5 to 87.8, *P *< 0.01); sE-selectin 24.1 ng/mL (5.5 to 42.7, *P *< 0.01); sICAM-1 135 ng/mL (67 to 202, *P *< 0.01); sVCAM-1 683 ng/mL (320 to 1046, *P *< 0.01).

There were a total of 52 patients with septic shock who in addition to the 0 hour draw had serial samples at 24, 48 and 72 hours. We compared biomarker levels in survivors (*n *= 43) to non-survivors (*n *= 9) (Figure 5). Using a linear mixed-effects model, adjusting for age, gender, and co-morbid burden, we found the following estimated mean differences in biomarker levels over time comparing the non-survivors to survivors: sFlt-1 366 pg/mL (95% CI: 218 to 514, *P *< 0.01); PAI-1 63.2 ng/ml (38.5 to 87.8, *P *< 0.01); sE-selectin 24.1 ng/mL (5.5 to 42.7, *P *< 0.01); sICAM-1 135 ng/mL (67 to 202, *P *< 0.01); and, sVCAM-1 683 ng/mL (320 to 1,046, *P *< 0.01).

## Discussion

The endothelium plays a key role in mediating vasomotor tone, leukocyte trafficking, permeability, and hemostasis (reviewed in [17,18]; Figure 1). Activation and dysfunction of the endothelium is characterized by increased permeability, vasodilation, recruitment of leukocytes, and a shift in the hemostatic balance towards the procoagulant side. Our findings in a group of moderately ill emergency department patients (mortality rate = 8%, 40% ICU admission rate) that sepsis severity is associated with increased circulating levels of sFlt-1, sICAM-1, sVCAM-1, sE-selectin and PAI-1 are consistent with the hypothesis that the endothelium is activated in sepsis.

Leukocyte trafficking across the endothelium involves a tightly regulated multistep process (reviewed in [19], Figure 1). Endothelial E-selectin and P-selectin regulate leukocyte rolling on the endothelium, whereas ICAM-1 and VCAM-1 are involved in firm adhesion. Many *in vitro *studies have demonstrated that activation agonists induce the mRNA and protein expression of these cell adhesion molecules. Expression levels are also increased in animal models of sepsis [20,21]. In contrast to animal models, there are currently no reliable assays for adhesion molecules in the intact endothelium of humans. In a recent proof-of-concept study, we showed the potential value of skin biopsies for assaying adhesion molecule expression in sepsis [21]. However, the protocol is invasive, and the data do not necessarily extrapolate to vascular beds outside the skin. A more common approach is to measure circulating levels of soluble adhesion molecule receptors as surrogate markers of endothelial activation. P- and E-selectin, ICAM-1 and VCAM-1 all undergo proteolytic cleavage of the extracellular region of the membrane-bound receptor [22-25] and levels of these soluble forms are increased in experimental and clinical sepsis [26-34]. Consistent with these published reports, our results show that sepsis is associated with elevated circulating levels of soluble ICAM-1, VCAM-1 and E-selectin. The levels were directly correlated with severity of illness and SOFA score, supporting the notion that the endothelium undergoes graded activation during the host response to infection.

The endothelium also balances hemostasis, which too, is deranged in sepsis (reviewed in [35]). Consistent with the results of previous studies [36-41], we have shown that PAI-1 levels are increased in severe sepsis, and that such levels correlate with the degree of severity. Since PAI-1 is largely restricted in its expression to endothelial cells, these findings add further support to the conclusion that the endothelium becomes increasingly activated during the host response.

Using animal models of sepsis, we have recently shown that VEGF plays an important role in mediating sepsis pathophysiology [20]. The biological plausibility of these findings is supported by the observation that VEGF signaling in endothelial cells results in an activation phenotype, including increased permeability, induction of cell adhesion molecules [42-44], the release of cytokines and chemokines, and the expression of procoagulant molecules [44]. VEGF binds to two receptors on the surface of endothelial cells, Flk-1 (also known as VEGFR2 or KDR) and Flt-1 (also known as VEGFR1). Flt-1 is also produced as a soluble receptor, sFlt-1, via alternative splicing of the precursor mRNA and functions as a decoy molecule, competing with membrane-bound Flt-1 for binding to VEGF. Indeed, we showed that the systemic administration of sFlt-1 (levels of approximately 20-fold over baseline) blocked sepsis morbidity and mortality in mice. Interestingly, endotoxin challenge in mice resulted in elevated (approximately five-fold) circulating levels of sFlt-1. We confirmed these observations in a small number of human patients with severe sepsis [4]. Together, these data suggested that sFlt-1 contributes to the systemic anti-inflammatory host response to infection. In the current study, we have extended these findings by showing that sFlt-1 is increased in patients with sepsis and that it is a superior marker of sepsis severity compared with the other markers tested.

Our findings add to the existing literature in important ways. First, with the exception of a study in which PAI-1 levels were measured in 840 patients with severe sepsis enrolled in the PROWESS trial [36], the current report includes the largest cohort of sepsis patients analyzed to date for soluble markers of endothelial activation. Second, the study is the only one that we are aware of that has included endothelial markers of both leukocyte adhesion and coagulation in the same population of patients. The finding that sFlt-1 levels correlate more closely with severity of illness and are a stronger predictor of organ dysfunction and mortality compared with soluble adhesion molecule receptors, IL-6, and lactate is novel. Moreover, the observation that multiple markers fail to provide additional information over single markers provides an impetus to focus a single diagnostic mediator in future prospective studies. Finally, the results of the current study convincingly validate our previous findings and demonstrate the promising value of sFlt-1 as a novel marker of sepsis morbidity and mortality.

### Limitations

This study has a number of important limitations. First, it was a convenience sample that may have suffered from selection bias. However, the population was selected to obtain a spectrum of severities as opposed to a consecutive sample of patients. Second, we primarily only analyzed blood from the initial draw, and except in the septic shock subset, did not follow biomarkers over time. Third, in our modeling, we adjusted for age, gender, and co-morbidity, but other important confounders may have affected our results. Fourth, circulating levels of endothelial biomarkers are only indirect measures of endothelial cell activation, and thus may not accurately reflect the degree, nature and site of activation of the intact endothelium. While we have selected representative biomarkers, others may still be more accurate. Fifth, we did not include a population of non-infected critically ill patients (for example, trauma patients) so we are unable to answer whether the endothelial cell changes are specific to sepsis, or broader markers of illness severity that would extend across disease states. Finally, our sample size is reasonable, but a larger study may have afforded the opportunity for more complete subset analysis. Both our sample over time analysis and mortality analysis was limited by a small sample size.

## Conclusions

The data presented here provide compelling evidence that sepsis in humans is associated with activation of the endothelium as evidence by increased levels of circulating biomarkers. We did not, however, test whether these changes were specific to sepsis, or whether endothelial cell activation occurs in critically ill patients with other insults such as trauma related inflammation; this is an important future study. Our results do support the hypothesis that the endothelium is a potential important diagnostic and therapeutic target in sepsis research.

## Key messages

• There is an association between markers of endothelial cell activation/dysfunction and severity of illness and organ dysfunction in sepsis.

• There is good correlation between biomarkers associated with endothelial cell activation suggesting a *net *endothelial response in sepsis.

• sFLT-1 shows promise as a novel prognostic marker in sepsis.

## Abbreviations

APACHE II score: acute physiologic and chronic health evaluation II score; AUC: area under the curve; BIDMC: Beth Israel Deaconess Medical Center; ED: emergency department; ICAM-1: soluble intercellular adhesion molecule; IL-6: Interleukin-6; PAI-1: plasminogen activator inhibitors -1; sFlt-1: soluble fms-like tyrosine kinase-1; SIRS: systemic inflammatory response syndrome; SOFA score: sequential organ failure assessment score; VCAM-1: soluble vascular cell adhesion molecule; VEGF: vascular endothelial growth factor.

## Competing interests

This project was funded in part by an investigator initiated research grant from Eli Lilly. Dr. Shapiro has received research grants from Hutchinson Technologies, Eli Lilly, and Inverness Medical and has received speaker's honorarium from Inverness Medical. Dr. Schuetz receives speaker's honoraria from BRAHMS's Diagnostics and Biomerieux Inc. Dr. Trzeciak receives research support from Ikaria and serves as a consultant to Spectral Diagnostics, but does not receive any personal remuneration from any commercial interest.

## Authors' contributions

NS and WA conceived of the project and oversaw all components of the project and manuscript preparation. MS and KY played a substantial role in data acquisition. PS, SP, AJ, ST and LN contributed substantially to data interpretation and analysis. All authors contributed to writing the manuscript and have given final approval of the version to be published.
